# The Relationship Between Extra-Administrative Workload, Emotional Exhaustion, and Work Engagement of Primary and Secondary School Teachers: Based on Multilevel Linear Model Analysis

**DOI:** 10.3390/bs15101405

**Published:** 2025-10-16

**Authors:** Zifeng Shen, Ruiming Lan, Xiaojie Su, Rong Lian, Yingying Zhang

**Affiliations:** 1 School of Psychology, Fujian Normal University, Fuzhou 350117, China; qbx20250071@yjs.fjnu.edu.cn (Z.S.); qbx20240065@yjs.fjnu.edu.cn (R.L.);; 2 Normal School, Urumqi Vocational University, Urumqi 830002, China; 3 Family Education Institute, Open University of Fujian, Fuzhou 350013, China; 4 International College of Chinese Studie, Fujian Normal University, Fuzhou 350117, China

**Keywords:** extra-administrative workload, emotional exhaustion, work engagement, primary and secondary school teachers, multilevel linear model

## Abstract

Primary and secondary school teachers in China bear a substantial additional administrative workload. However, no quantitative study has examined the effects of this workload on teachers. Drawing on the Job Demands–Resources model and Conservation of Resources theory, we examined the relationship between extra-administrative workload (school level), emotional exhaustion (personal level), and work engagement (personal level) by administering questionnaires and estimating a multilevel linear model. A total of 318 teachers from 51 primary and secondary schools participated in the study. The results indicated that (a) school-level extra-administrative workload significantly and positively predicted teachers’ emotional exhaustion, and (b) emotional exhaustion, in turn, significantly and positively predicted teachers’ work engagement. These findings provide an empirical basis for school management and policy formulation in primary and secondary education and offer practical guidance for promoting teachers’ mental health.

## 1. Introduction

Investigations conducted between 2020 and 2021 into Chinese primary and secondary school teachers’ extra-administrative workload revealed that a substantial portion of their daily responsibilities was unrelated to education or teaching (Song et al., 2021; X. Zhu & Liu, 2020). In October 2023, the suicide of a young teacher from Henan, who jumped from a building after being assigned an excessive number of non-teaching tasks, drew nationwide attention. Within China’s primary and secondary education system, the burden of excessive administrative duties on teachers continues today (T. Zhu & Wang, 2024).

These data indicate that primary and secondary school teachers in China are under substantial pressure as a result of extra-administrative workload. In this study, extra-administrative workload refers to the additional responsibilities that teachers must handle alongside their routine educational work, such as conducting various assessments inside and outside the school, completing forms unrelated to teaching, and managing inspections, copywriting tasks, and other administrative affairs (X. Wang, 2020). Primary and secondary school teachers constitute the backbone of basic education in China (Li et al., 2022; N. Wang et al., 2022). If these teachers are unable to focus on instruction and instead allocate most of their time to extra-administrative workload, it will be difficult to further improve the quality of teaching in primary and secondary schools in China. Moreover, the extent to which this heavy extra-administrative workload affects the emotional well-being and work engagement of primary and secondary school teachers remains unclear. Therefore, the present study primarily aims to investigate this issue.

Some studies have pointed out that the heavy burden on primary and secondary school teachers may have a series of negative effects, such as damage to teachers’ physical and mental health, reduced work effectiveness, and hindered professional development (Cai, 2023). Within research on the unreasonable burdens placed on primary and secondary school teachers in China, most studies have been literature reviews or simple survey studies (Cai, 2023; Fan & Feng, 2021; Song et al., 2021). There has been insufficient exploration of the impact of extra-administrative workload on primary and secondary school teachers. Accordingly, drawing on the job demands–resources model and conservation of resources theory, this study will explore the specific impact of extra-administrative workload on primary and secondary school teachers. Its purpose is to provide empirical evidence to support reducing extra-administrative workload for primary and secondary school teachers.

### 1.1. Application of the Job Demands–Resources Model in the Present Study

The Job Demands–Resources (JD-R) model points out that every occupation has unique factors that affect workers’ physical and mental health and their working conditions (Demerouti, 2025; Demerouti & Bakker, 2011). All these factors can be classified as work requirements and work resources (Demerouti et al., 2001; Gou et al., 2024). Work requirements are the primary predictors in the health-impairment process, and excessively high or prolonged work requirements can lead to health damage (Baka et al., 2023). Some studies have indicated that primary and secondary school teachers’ workload can positively predict emotional exhaustion (Schulze-Hagenest et al., 2023; H. Wang et al., 2025). Emotional exhaustion refers to a state in which individuals perceive that their emotional and related physiological resources have been depleted at work (Wright & Cropanzano, 1998).

First, extra-administrative workload, an additional work requirement that is not part of the original responsibilities of primary and secondary school teachers, will impose a heavy workload on teachers and consume considerable physical and mental resources. Second, this extra-administrative workload will occupy much of teachers’ working hours, leaving them insufficient time to prepare their daily lessons. This situation heightens instructional pressure on teachers. Faced with these pressures, teachers may experience negative emotions, such as anxiety and anger (Cavallari et al., 2024; H. Wang et al., 2023), thereby consuming their emotional resources and eventually leading to emotional exhaustion. Therefore, this study hypothesizes that the school-level extra-administrative workload will significantly and positively predict the emotional exhaustion of primary and secondary school teachers (H1).

### 1.2. Application of the Conservation of Resources in the Present Study

Conservation of Resources (COR) theory holds that when individual resources are lost or threatened, individuals will choose to conserve their resources to avoid further losses (Hobfoll, 2001). According to this theory, when primary and secondary school teachers are in a state of emotional exhaustion, they may invest fewer personal resources in their work—that is, lower their level of work engagement—in order to prevent additional depletion. Work engagement refers to a positive emotional and motivational state in which employees are energized, highly dedicated, and immersed in their work (Bakker & Albrecht, 2018). Research indicates a close relationship between individuals’ emotional exhaustion and their work engagement.

Results from studies that focus on company employees as participants and using variables as the central focus show that an individual’s emotional exhaustion significantly and negatively predicts his or her work engagement (Gillet et al., 2020; Sahi et al., 2022). Across both restaurant waitstaff and hotel service employees findings converge: subgroups with lower work engagement consistently report significantly higher emotional exhaustion, underscoring a robust inverse relationship between the two variables (Hou & Fan, 2024; Tetteh, 2024). This pattern may occur because emotionally exhausted individuals adopt negative coping strategies and retreat behaviors to protect their remaining resources, which leads to a decline in performance (Tan & Xu, 2023). Therefore, this study hypothesizes that the emotional exhaustion of primary and secondary school teachers will significantly and negatively predict their work engagement (H2).

A multilevel linear model (MLM) is a statistical analysis method. The most significant difference between MLM and traditional regression analysis lies in the treatment of variables at different levels. MLM considers how independent variables at high levels influence outcomes at the individual level. Traditional regression analysis places variables at the individual and organizational levels in the same regression model, thereby violating the assumption that independent variables are mutually independent (Van Dusen & Nissen, 2019; Woltman et al., 2012). MLM, on the other hand, possesses characteristics that render its model assumptions more realistic and its result interpretation more defensible (Liu & Meng, 2002). This approach addresses limitations inherent in one-level models that focus solely on school or teacher-level effects (Zhang, 2025). Therefore, this study will employ the MLM approach to examine relationships among extra-administrative workload (school level), teachers’ emotional exhaustion (individual level), and work engagement (individual level) in primary and secondary schools.

**Figure 1:**
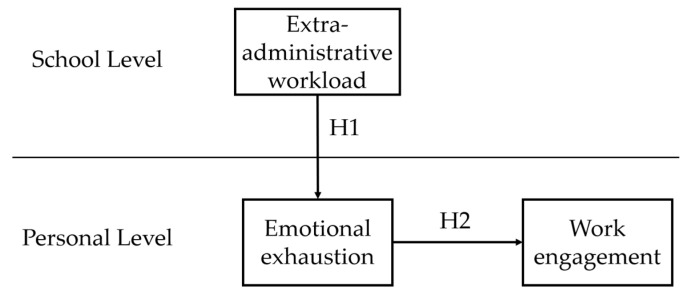
Hypothesis model.

In summary, based on the JD-R model and the COR theory, this study investigates the relationships between extra-administrative workload (school level), emotional exhaustion (personal level), and work engagement (personal level) in primary and secondary school teachers. To address these issues, we distributed questionnaires to primary and secondary school teachers to collect data and analyzed the relationships among the variables using MLM. The hypothetical model is shown in Figure 1.

Through this study, we can gain insights into the relationship between primary and secondary school teachers’ extra-administrative workload, emotional exhaustion, and work engagement. These findings support reducing the additional administrative workload assigned to primary and secondary school teachers, therefore enhancing their work engagement. Thus, enabling them to concentrate on teaching will, in turn, elevate the quality of primary and secondary school education.

## 2. Methods

### 2.1. Participants and Measurement Procedures

Maas and Hox (2005) pointed out that when using data with fifty groups and at least five samples in each group for MLM analysis, relatively unbiased estimation results can already be obtained. Therefore, data for the present study were collected in accordance with the recommendations of Maas and Hox.

Following this guideline and after receiving approval from the Research Ethics Committee of the School of Psychology, Fujian Normal University, we invited full-time primary and secondary school teachers across Fujian Province, China, to complete an anonymous online questionnaire hosted on wjx.cn from March to May 2024. Employing a convenience-plus-snowball strategy, we first contacted the headmasters we know and then asked cooperating headmasters to introduce us to additional schools.

Headmasters distributed the survey link to all teaching staff. When primary and secondary school teachers opened the questionnaire link, they first read an informed consent form displayed on the opening page. The informed consent form stated that participants’ responses would remain anonymous and confidential, and that they could withdraw at any time while completing the questionnaire. Only after teachers had read and agreed to these terms could they proceed to the formal questionnaire.

After data collection, we screened the data to eliminate invalid samples. We excluded cases that met either of the following criteria: (1) participants who displayed regular response patterns (e.g., always selecting the same option); or (2) schools with fewer than five valid participants, in which case all data from that school were discarded.

In this study, there were 408 initial data received. After screening, the final valid data amounted to 317, yielding an effective rate of 77.70%, encompassing 51 schools (comprising 34 primary schools, accounting for 66.67%, and 17 middle schools, accounting for 33.33%). Among the 317 teachers, 266 are women (83.91%), and 51 are men (16.09%). The oldest teacher is 57, the youngest is 21, and the average age is 32.78 (*SD* = 8.65). The maximum teaching experience is 35 years, the minimum teaching experience is 1 year, and the average teaching experience is 9.25 (*SD* = 9.67).

### 2.2. Research Tools

#### 2.2.1. Extra-Administrative Workload

This study used a self-developed questionnaire on extra-administrative workload for primary and secondary school teachers. The questionnaire was designed on the basis of recent research findings regarding extra-administrative workload among primary and secondary school teachers (Song et al., 2021; X. Zhu & Liu, 2020). The questionnaire consisted of five items, each focusing on extra-administrative workload (e.g., ‘responsible for the collection of various non-teaching-related forms’). The items were rated on a seven-point Likert scale, ranging from 1 (‘never’) to 7 (‘always’). Higher scores indicated a greater extent of extra-administrative workload undertaken by teachers.

In this study, SPSS (Version 26) and Mplus (Version 7.4) were used to examine the reliability and validity of the measurement instruments (the same applies hereafter). The correlation coefficients between each item and the total questionnaire score ranged from 0.64 to 0.77, and the items’ communalities ranged from 0.39 to 0.65, making the data suitable for principal component analysis. Factor extraction was performed using the eigenvalue-greater-than-one criterion and the direct oblimin rotation for principal component analysis. The results showed that one factor was extracted, explaining 52.49% of the cumulative variance, with item loadings ranging from 0.62 to 0.81, which indicates satisfactory construct validity. The specific content of each item, the total correlation coefficient, and the factor loadings are presented in Table 1. Further evaluation of the questionnaire’s validity was conducted using confirmatory factor analysis (CFA). The results of the CFA showed that the model fit well (*χ*^2^/*df* = 2.19, RMSEA = 0.06, CFI = 0.99, TLI = 0.97, SRMR = 0.02). In this study, the questionnaire’s Cronbach’s α coefficient was 0.77.

#### 2.2.2. Emotional Exhaustion

This study used the three item emotional exhaustion scale developed by Watkins et al. (2015) (e.g., ‘I feel emotionally drained from my work’), which has been applied to the Chinese teacher population (Luo et al., 2023). The questionnaire was scored using a seven-point Likert scale, with response options ranging from 1 (‘never’) to 7 (‘always’). A higher score indicates greater emotional exhaustion. In this study, Cronbach’s α coefficient of the questionnaire was 0.93.

#### 2.2.3. Work Engagement

The present study used the UWES-9 scale developed and revised by Schaufeli et al. (2006) (e.g., ‘I am enthusiastic about my job’), which has been employed in Chinese teacher populations (Jiang et al., 2023). The scale comprises three dimensions: vigor, dedication, and focus, with a total of nine items, and is scored on a seven-point Likert scale ranging from 1 (‘never’) to 7 (‘always’). Higher scores indicate greater work engagement. In this study, Cronbach’s α for the three dimensions ranged from 0.79 to 0.86, and Cronbach’s α for the total scale was 0.92.

### 2.3. Data Analysis

(1) Common-method-bias and CFA tests were performed using Mplus (Version 7.4).

(2) Multilevel modeling analysis, multiple regression analysis, descriptive and correlational analyses were conducted using SPSS (Version 26).

The configuration of MLM depends on the specific needs and data characteristics of a study. In line with our research objectives, we used MLM to test two models: the null model, the intercepts-as-outcomes model (Sommet & Morselli, 2021).

The null model was used to assess whether the data exhibited sufficient intra-group homogeneity and inter-group variability to justify MLM. The intercepts-as-outcomes model was used to test the effect of school level extra-administrative workload on teachers’ emotional exhaustion. Finally, the multiple regression model was used to examine how individual-level emotional exhaustion predicted work engagement.

## 3. Results

### 3.1. Common Method Bias Test

Variables were collected via self-report in this study. To control common-method bias, we implemented procedural controls by stressing the test’s confidentiality and anonymity to participants. Additionally, to examine the presence of common-method bias, Harman’s single-factor test was performed using a one-factor analysis with all questionnaire items as indicators. The results indicated that the model-fit indices were *χ*^2^/*df* = 11.80, RMSEA = 0.19, CFI = 0.61, TLI = 0.56, and SRMR = 0.15, demonstrating poor fit. Thus, there was no significant common-method bias in this study.

### 3.2. Descriptive Statistics of Each Variable

To test whether the variables are closely related and to prepare for the MLM analysis, a Pearson correlation analysis was conducted. The results are presented in Table 2. First, we report the scores of primary and secondary school teachers on the three variables. On the 7-point scales/questionnaire, teachers’ mean scores were *M* = 4.34, *SD* = 0.89 for extra-administrative workload, *M* = 4.21, *SD* = 1.34 for emotional exhaustion, and *M* = 4.05, *SD* = 1.11 for work engagement. All three means are close to the scale midpoint (4): extra-administrative workload and emotional exhaustion are slightly above the midpoint, indicating moderately elevated perceptions of workload and exhaustion; work engagement was close to the midpoint, placing teachers on the cusp of the moderate range (borderline moderate engagement).

Next, we report the Pearson correlation coefficients among the variables. As shown in Table 2, age is significantly negatively correlated with emotional exhaustion, and average daily working hours are significantly positively correlated with emotional exhaustion. Age and teaching experience are significantly positively correlated with work engagement. Therefore, age, average daily working hours, and teaching experience were used as control variables in the follow-up analysis. Extra-administrative workload is significantly positively correlated with emotional exhaustion and significantly negatively correlated with work engagement. The Pearson correlation between extra-administrative workload and work engagement was *r* = 0.12 (*R*^2^ = 0.014, ≈1.4%), which falls in the “very small” effect-size range (Funder & Ozer, 2019). Although the relationship is statistically detectable, its magnitude is minimal and likely of limited practical significance. Emotional exhaustion is significantly negatively correlated with work engagement. These Pearson correlation results indicate close relationships among the main variables and support the subsequent MLM analysis.

### 3.3. Basic Characteristics and MLM Analysis

The appropriateness of aggregating extra-administrative workload from the personal level to the school level—i.e., testing for intra-school consistency versus inter-school variability of the variables—needs to be assessed before conducting an MLM analysis. The mean value of R_wg_ for extra-administrative workload in this study was 0.81, which meets the judgmental criterion of R_wg_ > 0.70 (Zohar, 2000), thus allowing for the aggregation of individual level data to the school level and enabling MLM analysis.

#### 3.3.1. Predicting of School Level Extra-Administrative Workload on Emotional Exhaustion

In this study, we first conducted a null-model analysis of emotional exhaustion to assess the suitability of MLM analysis. The model is as follows (Model 1):

Individual level: Emotional exhaustion*_ij_* = *β*_0*j*_ + *r_ij_*

School level: *β*_0*j*_ = *γ*_00_ + *μ*_0*j*_

The analysis results (see Table 3) show that the within-school variance (*σ*^2^) was 1.50, while the between-school variance (*τ*_00_) was 0.31. The calculated ICC (1) was 0.17, exceeding the recommended threshold of 0.12 (James, 1982). This result indicates that 17% of the variation in emotional exhaustion in this study comes from different schools, making it suitable for MLM analysis.

We fitted a random-intercept model to examine the effect of school level extra-administrative workload on emotional exhaustion among primary and secondary school teachers. The model is specified as follows (Model 2):

Individual level: Emotional exhaustion*_ij_* = *β*_0*j*_ + *β*_1*j*_ (Age)*_ij_* + *β*_2*j*_ (Average daily working hours)*_ij_* + *r_ij_*

School level: *β*_0*j*_ = *γ*_00_ + *γ*_01_ (Extra-administrative workload)*_j_* + *μ*_0*j*_

*β*_1*j*_ = *γ*_10_ + *μ*_1*j*_

*β*_2*j*_ = *γ*_20_ + *μ*_2*j*_

The results (see Table 3) show that, after controlling for age and average daily working hours, school level extra-administrative workload significantly and positively predicted individual level emotional exhaustion (*γ*_01_ = 0.69, *t* = 7.07, *p* < 0.001). This result indicates that a one-unit increase in grand-mean-centered, group-level extra-administrative workload was associated with an increase of 0.69 units in expected emotional exhaustion after controlling for other terms in the model. Hypothesis 1 was supported. Table 3 also shows that, in this study, school level extra-administrative workload alone accounted for 93.55% of the between-school variance in emotional exhaustion among primary and secondary school teachers (calculated as (0.31 − 0.02)/0.31 ≈ 0.9355).

#### 3.3.2. Predicting of Emotional Exhaustion on Work Engagement

We fitted a multiple regression model to examine the effect of emotional exhaustion on work engagement among primary and secondary school teachers. The model is specified as follows (Model 3):

Individual level: Work engagement*_i_* = *β*_0_ + *β*_1_ (Age)*_i_* + *β*_2_ (Teaching experience)*_i_* + *β*_3_ (Emotional exhaustion)*_i_* + *r_i_*

**Figure 2:**
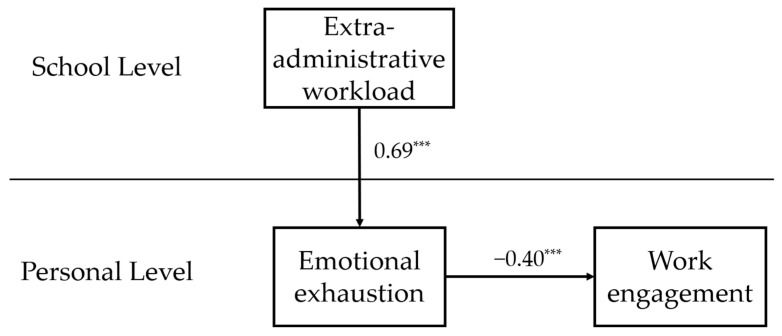
Overall model results; for brevity, control variables are omitted. Note: *** *p* < 0.001.

The results (see Table 3) show that, after controlling for age and teaching experience, emotional exhaustion significantly and negatively predicted work engagement among primary and secondary school teachers (*β*_2_ = −0.40, *t* = −11.30, *p* < 0.001). Hypothesis 2 was supported. Figure 2 presents the results of the overall model.

### 3.4. Result Summary

Grounded in the JD-R model and the COR theory, this study collected data via questionnaires and examined the relationships among extra-administrative workload (school level), emotional exhaustion (personal level), and work engagement (personal level) of primary and secondary school teachers using correlation, MLM and multiple regression analyses. Our main findings are as follows:

Results show that school-level extra-administrative workload significantly and positively predicts primary and secondary school teachers’ emotional exhaustion. This suggests that, in schools with a higher volume of such workload, teachers’ emotional exhaustion may be elevated. Emotional exhaustion, in turn, significantly and negatively predicts teachers’ work engagement. This suggests that the higher the level of emotional exhaustion, the lower teachers’ level of work engagement may be.

## 4. Discussion

### 4.1. Predictive Role of School-Level Extra-Administrative Workload on Emotional Exhaustion Among Primary and Secondary School Teachers

The result of this study on the relationship between school-level extra-administrative workload and emotional exhaustion was similar to previous findings (Schulze-Hagenest et al., 2023; H. Wang et al., 2025). According to the JD-R model (Demerouti, 2025; Demerouti & Bakker, 2011), school level additional administrative work can be understood as an organizational demand: it occupies teachers’ limited time for lesson preparation, instruction, and recovery, consumes excessive psychological resources of elementary and secondary school teachers, and thereby affects teachers’ emotions.

Surveys have shown that in 2018, the percentage of daily time spent on ex-tra-administrative workload exceeded the percentage spent on teaching work (X. Wang, 2020), placing considerable pressure on teachers (X. Zhu & Liu, 2020). Moreover, the more work-related pressure an individual faces, the more energy they lose (Tan & Xu, 2023). Because they continuously face extra-administrative duties, primary and secondary school teachers deplete their physiological and psychological resources without adequate recovery, ultimately leading to emotional exhaustion.

### 4.2. Predictive Role of Emotional Exhaustion on Work Engagement Among Primary and Secondary School Teachers

The result of this study on the relationship between emotional exhaustion and work engagement was similar to previous findings (Karahan Kaplan et al., 2024; Moreno-Martínez & Sánchez-Martínez, 2025). Task-related fatigue and other feelings of exhaustion are the two primary characteristics of emotional exhaustion (Maslach & Jackson, 1981; Zheng et al., 2015). Thus, individuals experiencing high levels of emotional exhaustion often struggle to feel energized at work and find it difficult to concentrate on necessary tasks. Moreover, according to the COR theory (Hobfoll, 2001), when teachers’ personal resources are depleted and not replenished and they are in a state of emotional exhaustion, they may adopt negative coping strategies and withdrawal behaviors to conserve their remaining resources. This, in turn, may lead to reduced work engagement.

Research has indicated that when individuals exhibit high levels of career involvement (Schaufeli et al., 2008), they may simultaneously display both high engagement and high exhaustion. In the present study, some primary and secondary school teachers might have maintained high levels of work engagement despite elevated emotional exhaustion. This pattern may reflect China’s culture of teacher dedication, which is reflected in two aspects. First, traditional Chinese values frequently construe teaching as a noble vocation that emphasizes selfless devotion and responsibility. Second, under current educational structures and prevailing societal expectations, teachers often encounter heavy workloads and substantial pressures. Despite these pressures, a strong professional mission and concern for students may nonetheless compel teachers to remain engaged even when emotionally depleted.

However, such care and dedication among primary and secondary school teachers should not be construed as a sustainable long-term strategy to maintain their work engagement. Instead, school administrators and policymakers should prioritize reducing teachers’ non-instructional administrative workload and optimizing work structures and support systems to foster a more focused and sustainable teaching environment. These structural changes may be more effective than relying on individual dedication to mitigate the adverse effects of emotional exhaustion.

### 4.3. Research Contributions and Recommendations

#### 4.3.1. Theoretical Contributions

Grounded in the JD-R model and COR theory, this multilevel study demonstrates that school level extra-administrative workload significantly predicts higher emotional exhaustion and, in turn, lower work engagement. By isolating a demand located at the school level, rather than at the classroom or individual level, the findings extend JD-R scholarship to a cross-level demand-strain process and corroborate COR’s assertion that contextual resource loss erodes personal energy.

#### 4.3.2. Practical Recommendations

Based on the results of this study, we make the following recommendations to maintain teachers’ mental health and occupational enthusiasm. (1) Primary and secondary schools that require extra-administrative work should recruit staff members who specialize in handling administrative tasks rather than assign such work to teachers. (2) Primary and secondary schools should avoid blindly expanding the scope of inspection tasks assigned by higher education departments. When an education department needs to inspect the quality of learning operations, it should provide an inspection checklist to standardize the content and process of inspection; any items not included in or approved by the checklist must not be implemented (X. Wang, 2020). (3) Primary and secondary schools should offer dedicated mental-health services for teachers. In the current environment, teachers’ extra-administrative workload continues to increase—a trend that is highly detrimental to their mental health and occupational enthusiasm. Schools should therefore not only reduce teachers’ extra-administrative workload but also help them mitigate its negative effects so that teachers feel rejuvenated.

### 4.4. Research Limitation and Prospects

The current study has the following limitations: (1) it employs a cross-sectional research design; therefore, it cannot verify causal relationships between variables. In the future, a longitudinal research design will be employed to better elucidate causal relationships between the independent and dependent variables. (2) It draws participants from a relatively homogeneous region, thereby limiting the generalizability of the results to a broader population. Future studies will expand the sampling area to enhance the study’s ecological validity.

## 5. Conclusions

The findings suggested that school-level extra-administrative workload significantly and positively predicts emotional exhaustion among primary and secondary school teachers, which in turn significantly and negatively predicts their work engagement.

These results highlight the need for primary and secondary schools to reduce the extra administrative work assigned to teachers.

## Figures and Tables

**Table 1:** Detailed Content of the Questionnaire on Additional Administrative Workload.

Specific Items	Total Correlation Coefficient	Communalities	Item Loading
1. Preparing for inspections unrelated to teaching	0.71 ***	0.53	0.73
2. Writing materials unrelated to teaching	0.75 ***	0.63	0.79
3. Attending training sessions unrelated to teaching	0.66 ***	0.39	0.62
4. Participate in various non-teaching training programs	0.64 ***	0.43	0.66
5. Responsible for collecting forms unrelated to teaching	0.77 ***	0.65	0.81

Note: *** *p* < 0.001.

**Table 2:** Description Statistics for Each Variable and Their Corresponding Analysis Results.

	1	2	3	4	5	6
1 Age	—					
2 Teaching experience (year)	0.97 ***	—				
3 Average daily working hours	0.06	0.05	—			
4 Extra-administrative workload	0.05	0.05	0.15 **	—		
5 Emotional exhaustion	−0.12 *	−0.10	0.14 **	0.50 ***	—	
6 Work engagement	0.26 ***	0.25 ***	0.03	−0.12 *	−0.55 ***	—
*M*	32.78	9.25	7.23	4.34	4.21	4.05
*SD*	8.65	9.67	2.62	0.89	1.34	1.11

Note: *n* = 317; ‘—’ not applicable; * *p* < 0.05, ** *p* < 0.01, *** *p* < 0.001.

**Table 3:** MLM Analysis Results.

	Model	Model 1	Model 2	Model 3
	Dependent Variable	Emotional Exhaustion	Emotional Exhaustion	Work Engagement
Fix effect	Person Level			
	Age	—	−0.02	0.01
	Teaching experience	—	—	0.01
	Average daily working hours	—	0.05	—
	Emotional exhaustion	—	—	−0.40 ***
	School Level			
	Extra-administrative workload	—	0.69 ***	—
Random effect	*σ* ^2^	1.50	1.50	0.69
	*τ*_00_	0.31	0.02 ^(b)^	—
Explained variance ^(a)^		—	0.29	0.28
Model fit	−2 Restricted Log Likelihood	1071.59	1036.28	800.37
	*AIC*	1075.59	1040.28	802.37
	*BIC*	1083.10	1047.78	806.12

Note: ^(a)^ Proportion of variance in the dependent variable explained by the primary independent variables in each model (control variables excluded). ^(b)^ Inclusion of control variables reduced the estimate below the software’s computable range; the reported value therefore refers to the model with only extra-administrative workload; ‘—’ not applicable; *** *p* < 0.001.

## Data Availability

Limited data can be supplied upon reasonable request to the corresponding author.
